# Supplementary material for: Bridging the gap between atomically thin semiconductors and metal leads
Source: Nat Commun. 2022 Apr 1;13:1777. doi: 10.1038/s41467-022-29449-4 (PMC8976069; doi:10.1038/s41467-022-29449-4)
Supplement: Supplementary file 1 — Supplementary Information [file 41467_2022_29449_MOESM1_ESM.pdf]

Supplementary Information for

Bridging the gap between atomically thin semiconductors and metal leads

Supplementary Figures:

- Supplementary Figure 1: Control device of 3L-MoS_2 by direct edge contacts
- Supplementary Figure 2: Sample fabrication steps
- Supplementary Figure 3: EELS analysis of the LBD contact region
- Supplementary Figure 4: Raman spectroscopy of the LBD
- Supplementary Figure 5: 1L-MoS_2 FET by LBD contacts
- Supplementary Figure 6: ACSTEM image analysis
- Supplementary Figure 7: Extended data of the 5L-WSe_2 FET using LBD contacts
- Supplementary Figure 8: Reproducibility and practicality of LBD contacts in TMDSCs
- Supplementary Figure 9: Lead-dependent polarity of WSe_2 FETs
- Supplementary Figure 10: LBD-induced metallicity in TMDSC monolayers
- Supplementary Figure 11: DFT-calculated work function of 1T MoS_2 nanoribbon
- Supplementary Figure 12: Transmission line experiments
- Supplementary Figure 13: Control experiments showing no e-beam artefacts in our atomic imaging
- Supplementary Figure 14: Detailed transport data of the Hall bar WSe_2 devices
- Supplementary Figure 15: Room-temperature contact resistance extraction

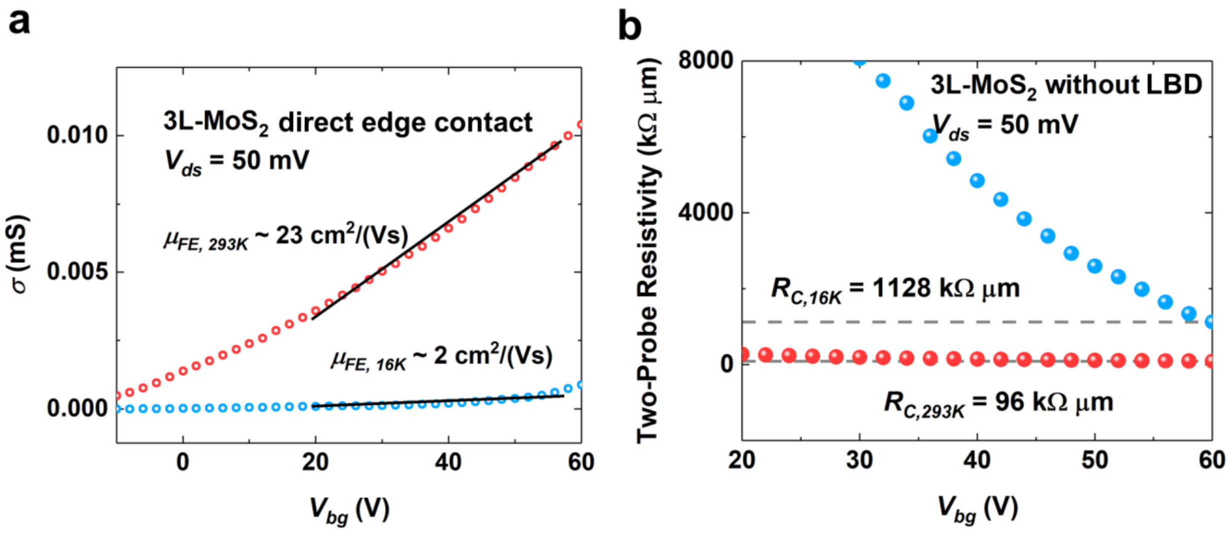

Supplementary Figure 1: Control device of 3L-MoS_2 by direct edge contacts. (a) Field-effect mobility and **(b)** contact resistance at room and cryogenic temperatures. The direct electrical interfacing between metal leads and semiconducting MoS_2 edges causes severe Fermi-level pinning and Schottky barriers, as the band diagram in Figure 1 b shows, which degrade the FET performance at cryogenic temperatures. As shown in Supplementary Figure 1, the channel conductance was low with four-order smaller mobilities and larger contact resistance than those utilizing LBD contacts reported in Figure 4. The device performance becomes worse at lower temperatures as the main carrier-injection mechanism in the contact interface shifts from the thermionic emission to the tunneling across Schottky barriers.

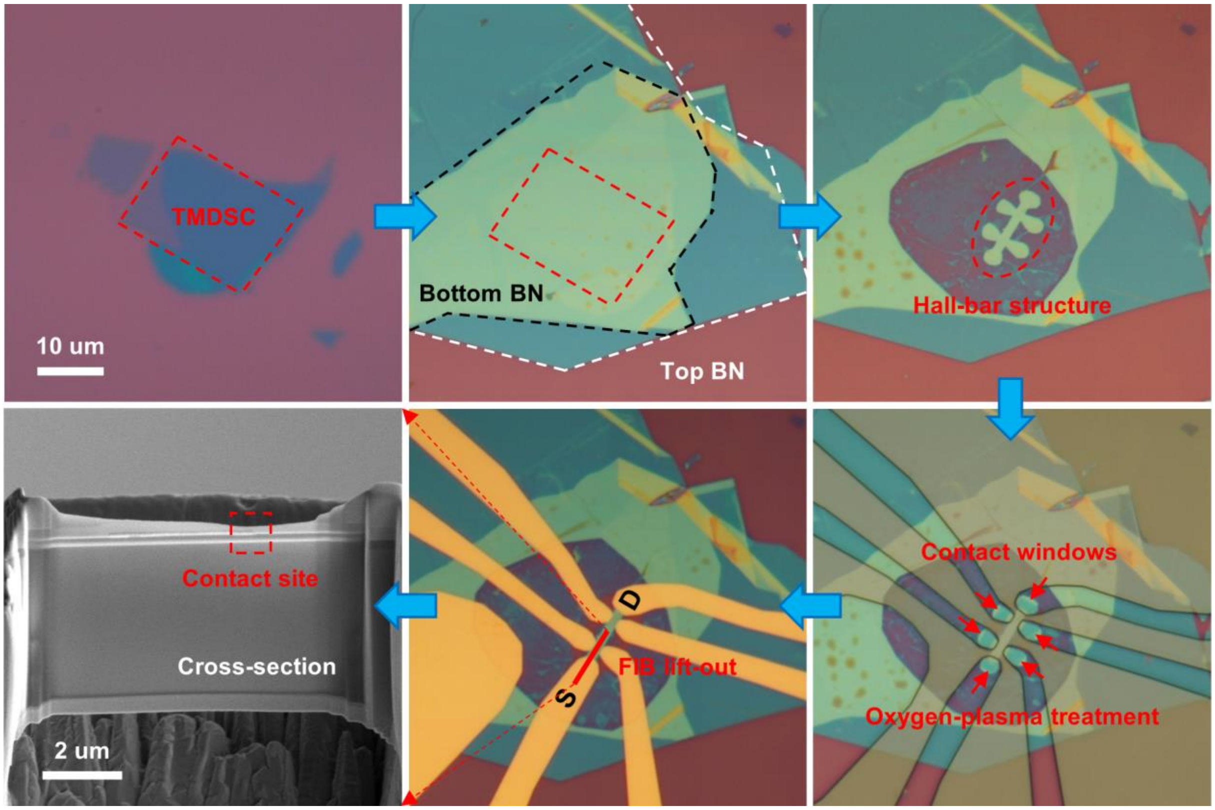

Supplementary Figure 2: Sample fabrication steps. Optical images showing the step-by-step FET fabrication by the LBD method: mechanical exfoliation; BN encapsulation by the dry transfer technique; Hall-bar shaping; contact windows by the referenced RIE processes; soft oxygen-plasma treatment; metal lead deposition by the electron-beam evaporation. After electrical measurements, cross-section samples from one of the main carrier-injection contacts were prepared by FIB techniques as shown in the SEM view.

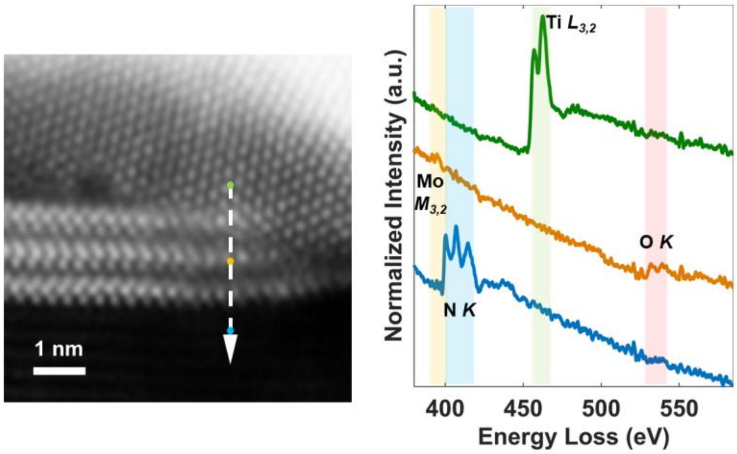

Supplementary Figure 3: EELS analysis of the LBD contact region. An EELS line-scan was performed across the metal/LBD/BN heterostructure as marked in the left ADF image. After aligning the energy-loss to the absolute energy scale according to the simultaneously recorded zero-loss peaks, the normalized spectra showing characteristic core-loss edges were plotted along the scan direction in the right panel. The oxygen K edge can be identified from the power-law background in the LBD region, suggesting the oxygen substitution.

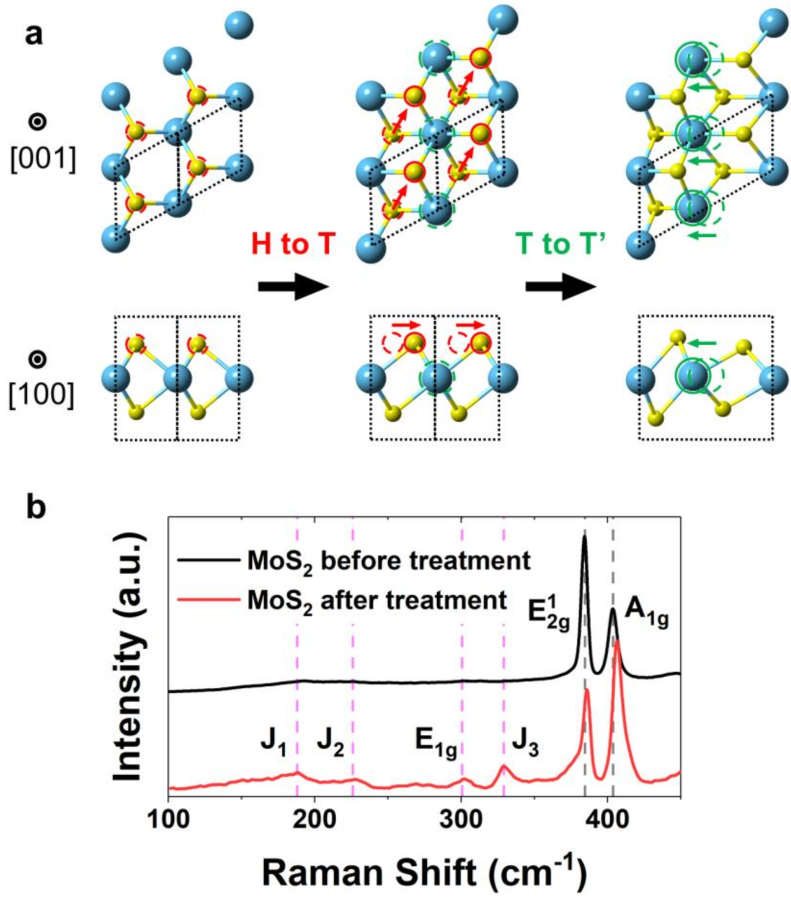

Supplementary Figure 4: Raman spectroscopy of the LBD. (a) Atomic structure transformation by TMDSC bond rearrangements (TM stands for transition metals and cyan balls in models; C stands for chalcogen atoms and yellow balls in models). Theoretically, only a slight sliding of top-layer chalcogen atoms along the <110> direction as shown by red arrows is necessary for the coordination change of [TM-C_6] polyhedra from the trigonal-prismatic coordination (H) to the octahedral one (T). Then a small shift of metal atoms along the <120> direction as shown by green arrows will lead to the distorted T structure (T’). Black parallelograms illustrate the unit cells. **(b)** Raman measurements on MoS_2 before and after the soft oxygen-plasma treatment. We conducted the Raman experiment during the fabrication of top contacts using a micro Raman system (model InVia of Renishaw), whose spot size is about 5 μm in diameter. The laser power during

measurements was kept below 100 μW to avoid any possible sample damage and excessive heating. The silicon Raman mode at 520 cm^{-1} was used for the system calibration before spectra acquisition. Long-time (~30s) signal acquisition at each ~1.8x2.3 μm^2 phase-transitioned contact window was adopted and further integrated from six contact windows in the same device to produce the final spectra of higher signal-to-noise ratio. The blue shift and broadening of characteristic Raman modes, i.e. E_{2g} (in-plane vibration) and A_{1g} (out-of-plane vibration), indicate the occurrence of bonding distortion, which is consistent with the literature about Raman spectroscopy of lattice distortion.[1] There are also some emergent soft modes in the Raman spectrum of treated MoS_2 being assigned to the J_1, J_2 and J_3 Raman modes of 1T and 1T' MoS_2 structures as marked by magenta dashed lines, which originate from the octahedral coordination after the LBD process.[2]

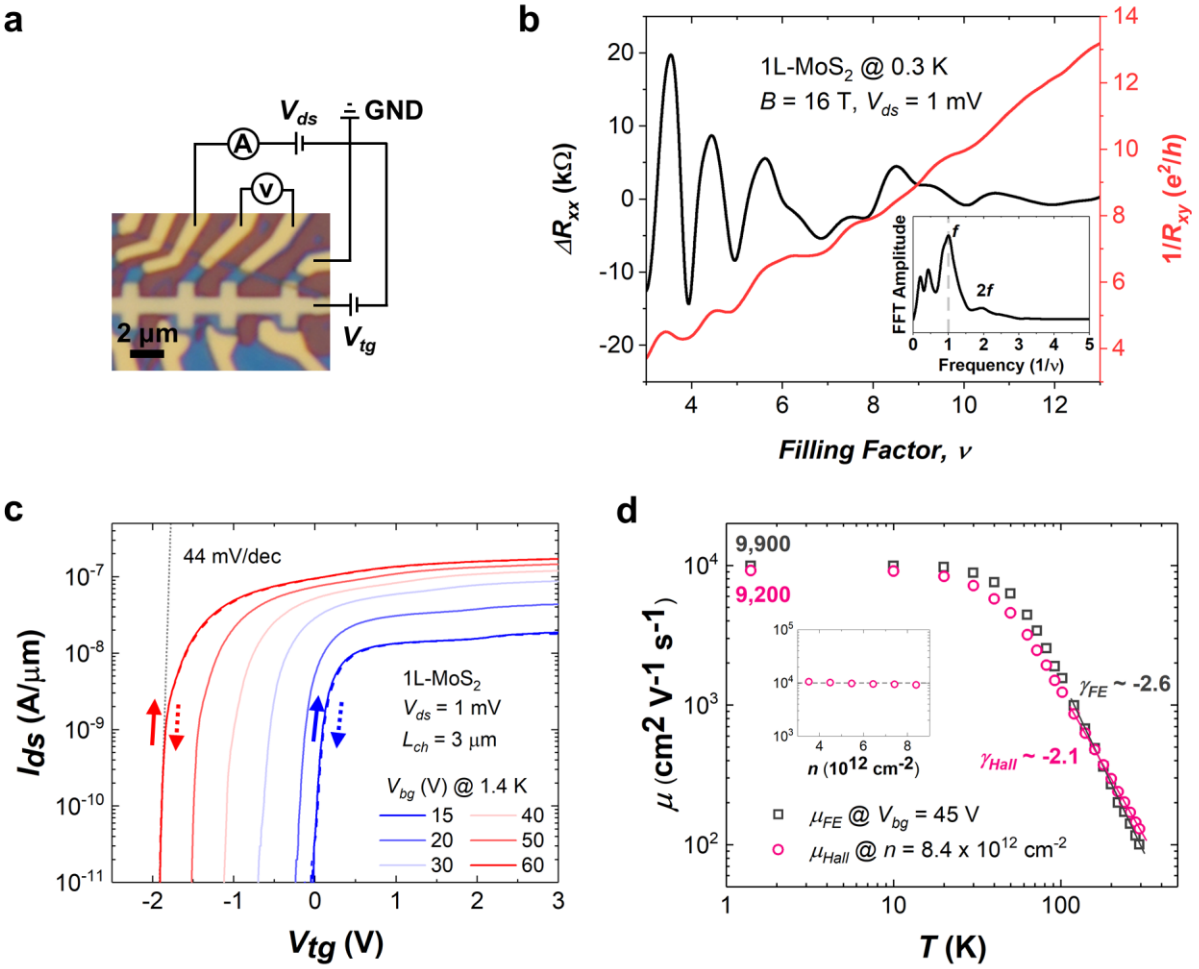

Supplementary Figure 5: 1L-MoS_2 FET by LBD contacts. (a) Optical image of a dual-gated 1L-MoS_2 FET utilizing the LBD top contact method. **(b)** Quantum transport measurements showing prominent R_{xx} oscillation and R_{xy} quantization. The inset is the fast Fourier transform (FFT) amplitude of R_{xx} versus filling factors, indicating the principal oscillation period of $\Delta\nu$=1. **(c)** Top-gated transfer curves with V_{bg} varied from 15 to 60 V. Negligible hysteresis is shown for both upper and lower V_{bg} limits. **(d)** Temperature-dependent characteristics of the field-effect and Hall mobilities. The Hall mobility increases slightly with lowered carrier concentrations at 1.4 K as the inset shows.

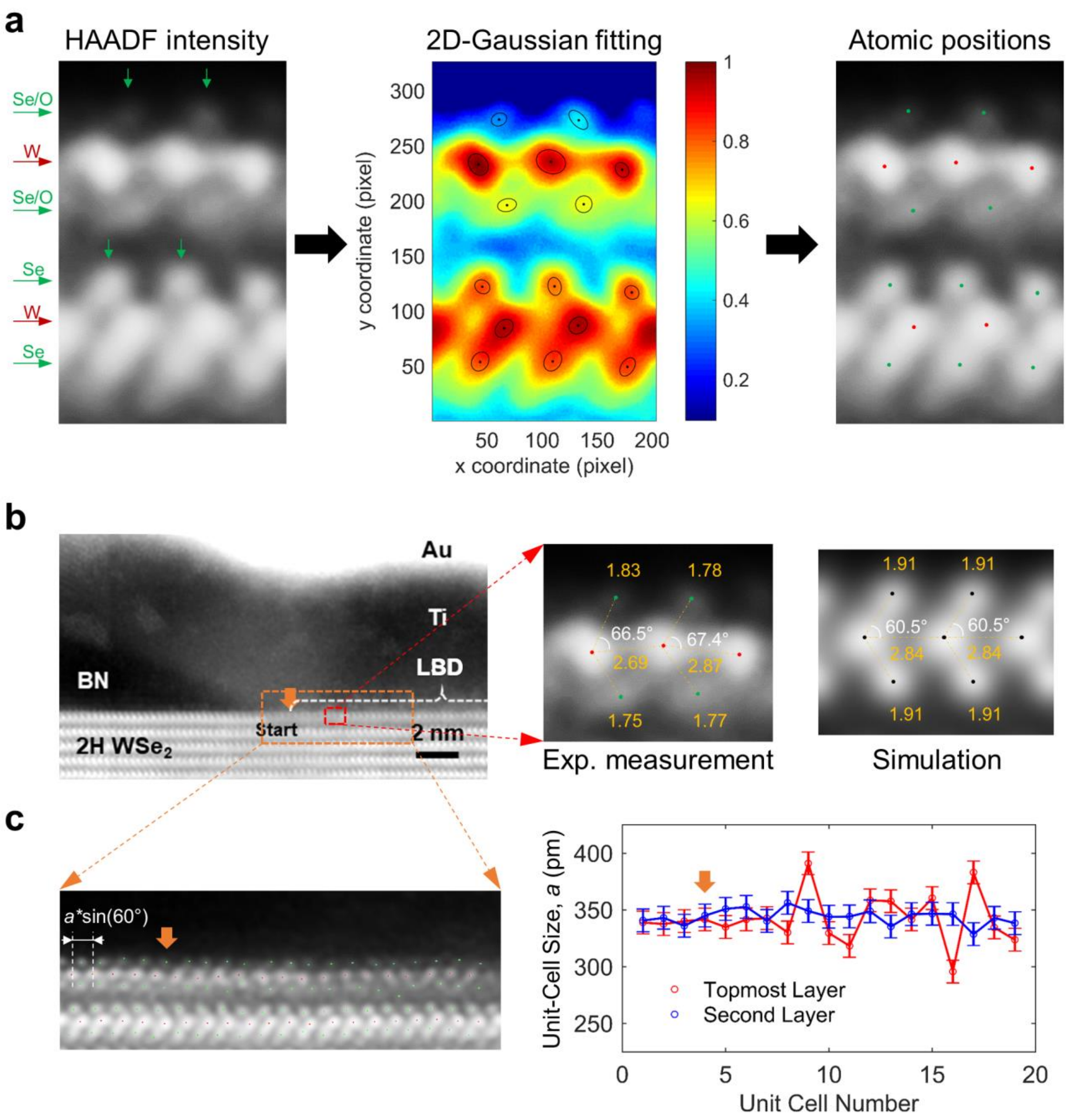

Supplementary Figure 6: ACSTEM image analysis. (a) The data-processing procedures to determine atomic positions, where red and green dots are the fitted W and Se atomic positions, respectively. The atomic positions were precisely determined by the 2D-gaussian fitting of normalized HAADF intensities of each atomic column. **(b)** Quantitative analysis of the structure distortion by bond lengths and angles. The WSe_2 layer at the contact region distorted locally by

the shrinking of first W-W bond length and the elongating of following one, together with shortened W-Se/O bond lengths and stretched Se-W-Se angles, when compared to the simulated standard structure (equal W-W, W-Se spacings and Se-W-Se angles in all unit cells). **(c)** The variation of distortion across a wider range and in different layers of WSe_2 is evaluated by calculating the lattice parameter, *a*, in each unit cell. The error bars are defined from the fitting accuracy of atomic positions. It can be seen that the distortion starts right inside the contact region, as marked by orange arrows, showing locally shrunk and elongated unit-cell sizes. But the second layer is protected by the topmost layer and its unit-cell sizes remain relatively constant as that in the standard structure. Such single-layer-depth distortion explains the good controllability of the soft oxygen plasma treatment from monolayer to few-layer samples, since the LBD self-saturates in the topmost layer and serves as the carrier-injection bridge no matter of the sample thicknesses.

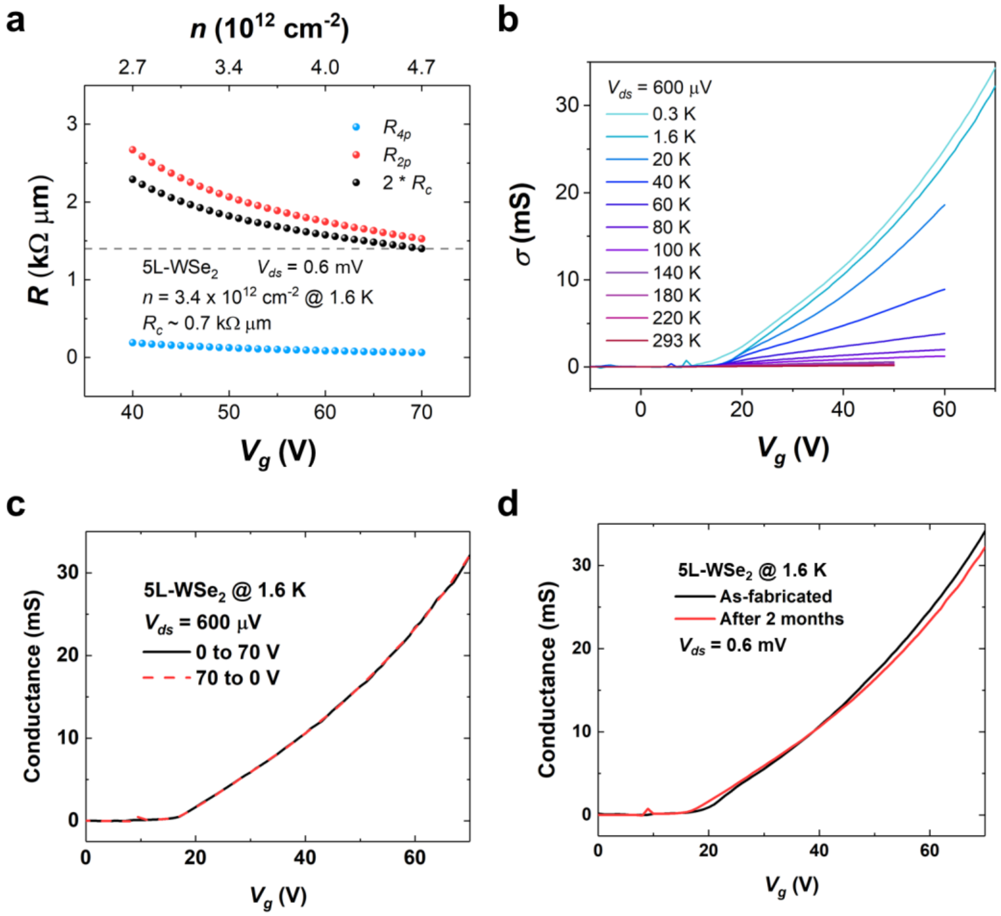

Supplementary Figure 7: Extended data of the 5L-WSe_2 FET using LBD contacts. (a) Extraction of contact resistance by four-probe measurements. **(b)** Temperature-varied transfer curves, from which field-effect mobilities at each temperature can be calculated. **(c)** Forward- and backward-scans of the channel conductance. **(d)** Transfer curves of the same 5L-WSe_2 FET right after the fabrication and after 2-month storage. The small shift of the threshold voltage may be due to the annealing effects when removing the sample from the cryogenic station after the first-round test.

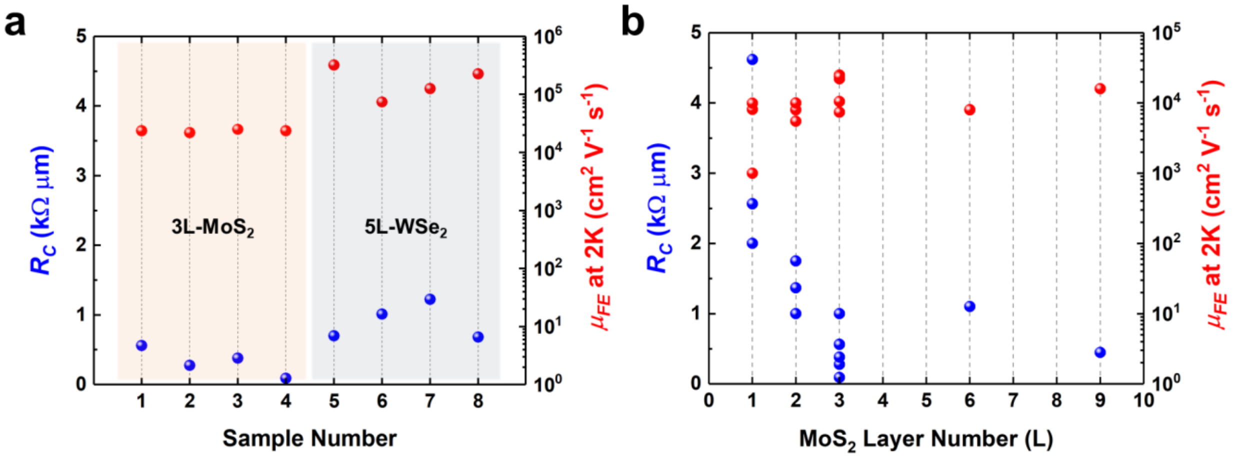

Supplementary Figure 8: Reproducibility and practicality of LBD contacts in TMDSCs. (a) Contact resistance and field-effect mobility of multiple back-gated 3L-MoS_2 and 5L-WSe_2 FETs using the LBD contact strategy. The high-quality contact is reproducible once suitable conditions are set up. The device deviation is reasonable considering the fabrication error by human handling and laboratory facilities. We also notice that WSe_2 FETs show a larger device-to-device variation, which may be attributed to the higher mixed state of octahedral derivatives than the situation of MoS_2. **(b)** Device performance of multiple MoS_2 FETs of different layer numbers. Both the achievable charge carrier mobility and contact resistance depend strongly on the channel thickness, i.e. thinner channel leads to suppressed mobility and higher contact resistance. The dramatically increased contact resistance and lowered carrier mobility for monolayers may be due to the weaker screening effect and thus increased scattering by impurity in the atomically thin channels.

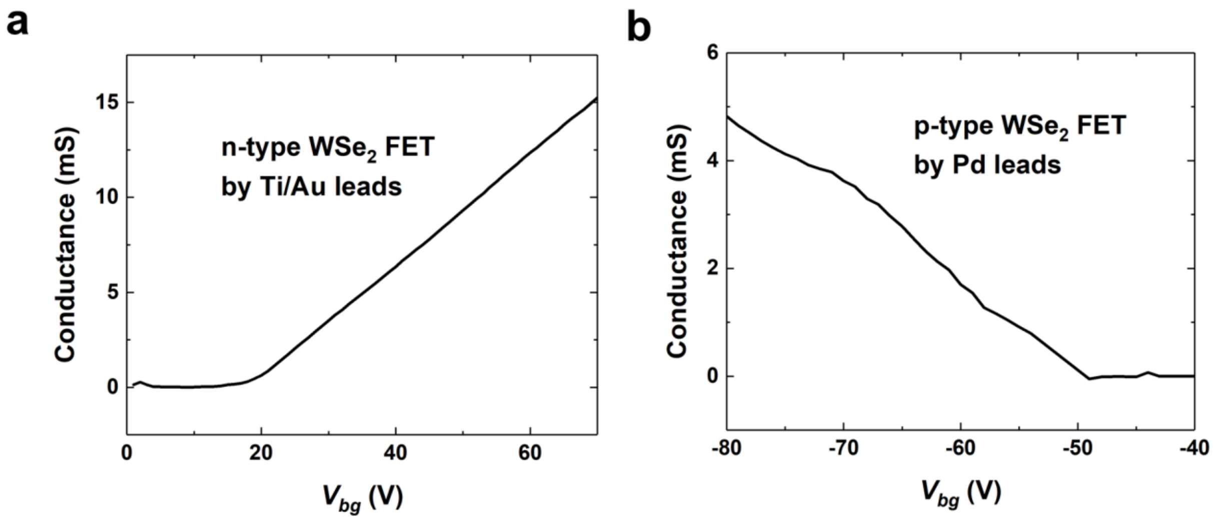

Supplementary Figure 9: Lead-dependent polarity of WSe_2 FETs. Low-temperature transfer curves of few-layer WSe_2 FETs fabricated by the same LBD contact method but with **(a)** Ti/Au and **(b)** Pd leads, respectively.

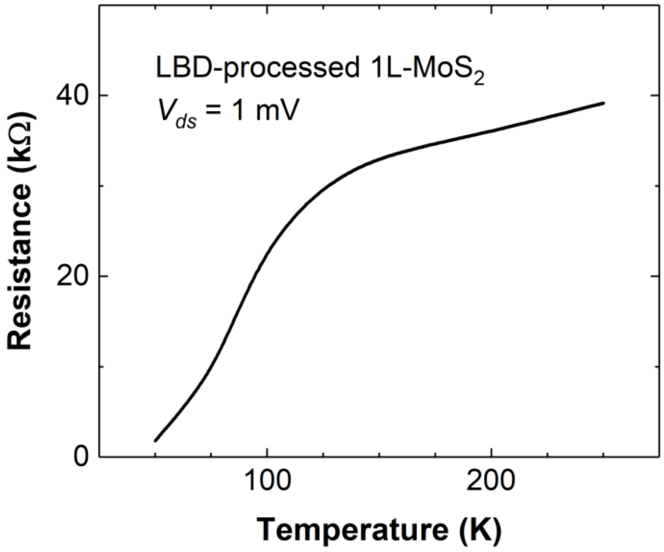

Supplementary Figure 10: LBD-induced metallicity in TMDSC monolayers. The resistance of 1L-MoS_2 after treated by the same soft oxygen-plasma process as fabricating LBD contacts was measured at varied temperatures. It can be seen that the resistance of processed 1L-MoS_2 shows the metallic behavior by continuously decreasing when the temperature lowers. The sharper drop of resistance below 150 K may originate from the de-saturation of those dispersed DOS near the Fermi level, suggesting semi-metallic characteristics.

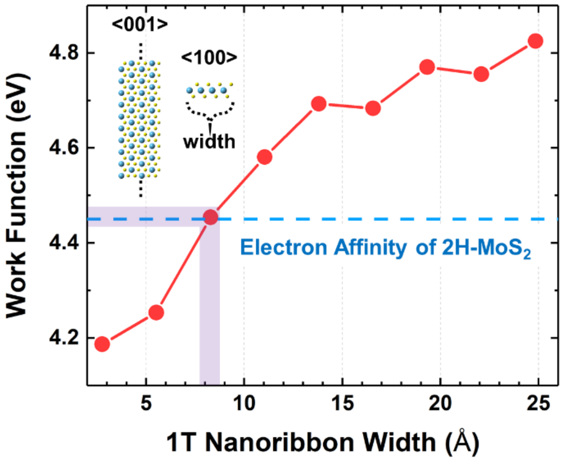

Supplementary Figure 11: DFT-calculated work function of 1T MoS_2 nanoribbon. The work function of bulk 1T MoS_2 mismatches the electron affinity of 2H MoS_2, leading to the Schottky-barrier-limited contact and larger contact resistance.[3] When the size of 1T phase decreases (~1 nm in our cases), its work function lowers due to quantum confinement effects and match the electron affinity of 2H MoS_2 at ~1 nm width, which reduces the Schottky barrier height and thus the achievable contact resistance.

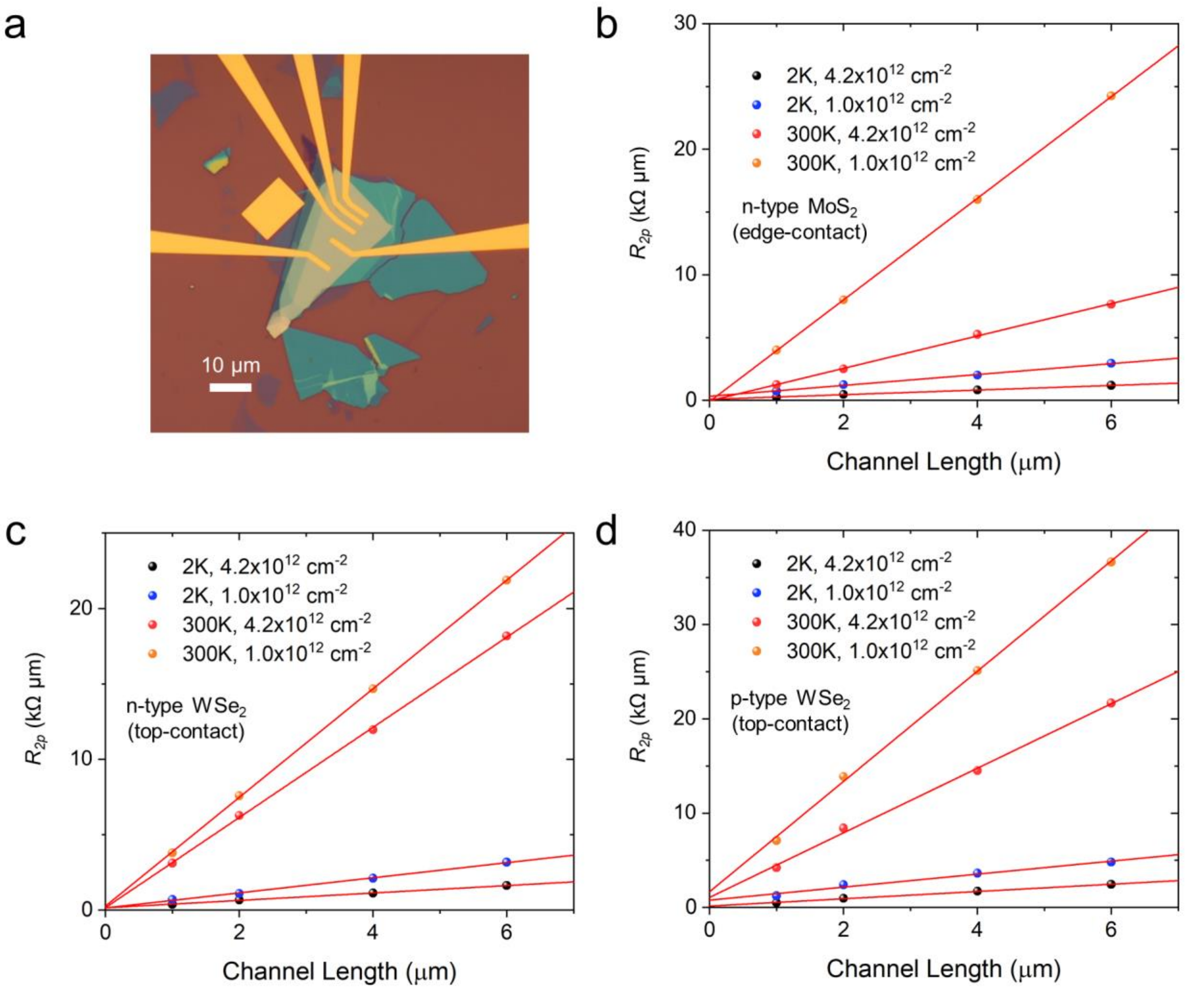

Supplementary Figure 12: Transmission line experiments. (a) Optical image of a typical transmission line device. Transmission line measurements of **(b)** edge-contacted n-type MoS_2, **(c)** top-contacted n-type WSe_2 and **(d)** top-contacted p-type WSe_2 at varied channel carrier densities and temperatures.

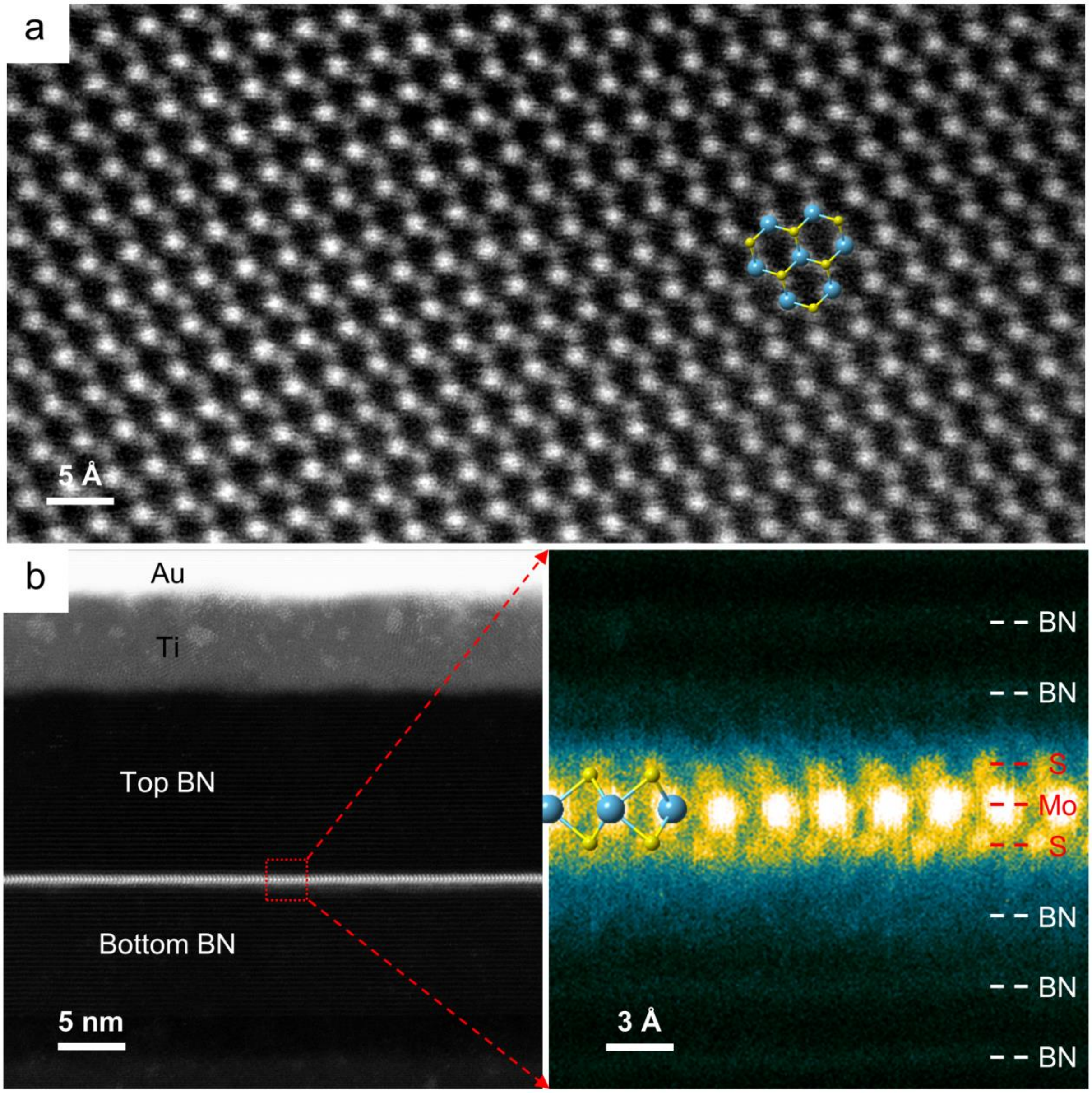

Supplementary Figure 13: Control experiments showing no e-beam artefacts in our atomic imaging. **(a)** Plane-view and **(b)** cross-section imaging of the pristine monolayer MoS_2 under the same conditions as the atomic imaging of device contact regions.

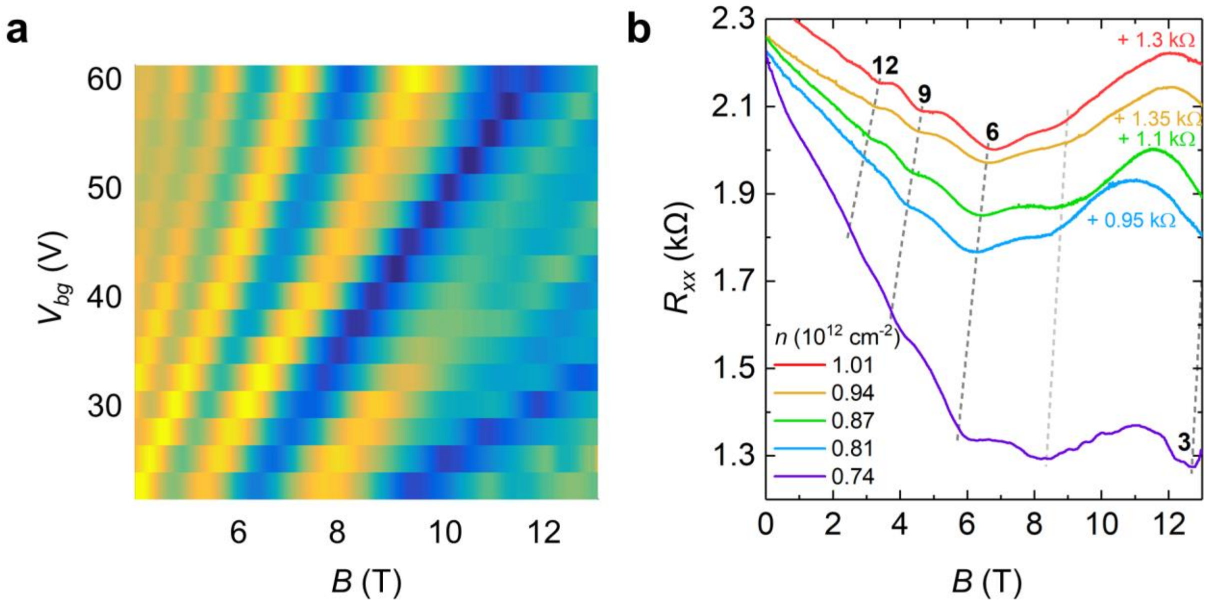

Supplementary Figure 14: Detailed transport data of the Hall-bar WSe_2 devices. (a) Color map of the R_{xx} of 5L-WSe_2 FET, showing the Landau quantization along varied gate voltages (carrier density) and magnetic field strengths. **(b)** SdH oscillations at low carrier densities, showing the achievable lowest integer Landau level of Q valley.

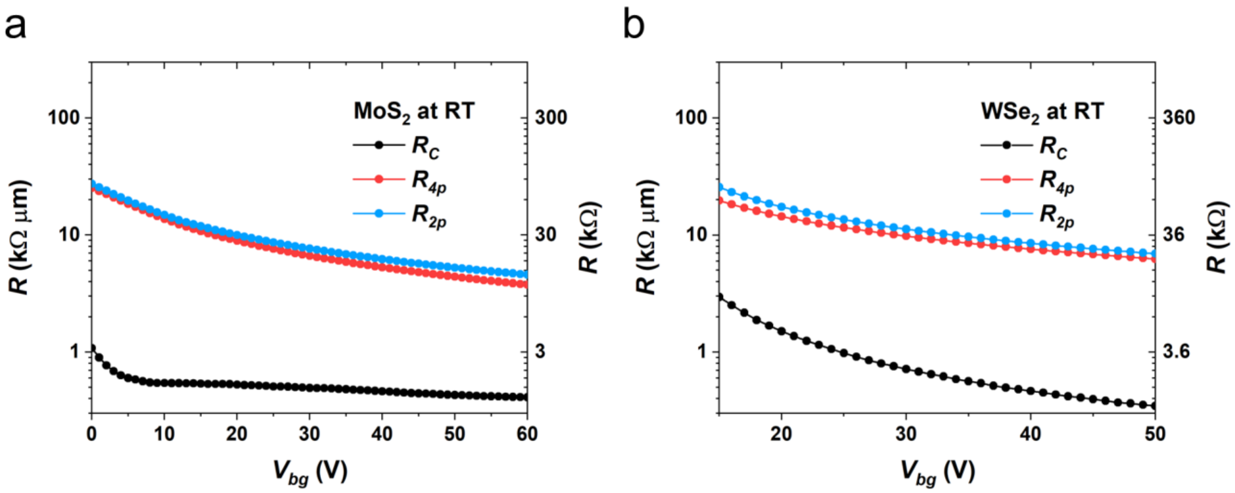

Supplementary Figure 15: Room-temperature contact resistance extraction. The extraction of contact resistance by comparing the two- and four-terminal resistance of LBD-contacted **(a)** MoS_2 and **(b)** WSe_2 FETs at room temperature, where both resistance values in kΩ·μm and kΩ units are plotted in the logarithmic scale. The four-probe resistance is scaled by L_{out}/L_{in} as described in the Methods section.

Supplementary References:

1 Nguyen, T. H. *et al.* Raman spectroscopic evidence of impurity-induced structural distortion in SmB_6. *Journal of Raman Spectroscopy* **50**, 1661-1671 (2019).

2 Nayak, A. *et al.* Pressure-dependent optical and vibrational properties of monolayer molybdenum disulfide. *Nano Letters* **15**, 346-353 (2015).

3 Fan, Z. *et al.* In-plane schottky-barrier field-effect transistors based on 1T/2H heterojunctions of transition-metal dichalcogenides. *Physical Review B* **96**, 165402 (2017).
